# High-resistance inspiratory muscle strength training: a promising strategy for improving vascular health in chronic kidney disease

**DOI:** 10.3389/fphys.2025.1582777

**Published:** 2025-04-25

**Authors:** Stephanie Lapierre-Nguyen, Tyler Buffington, Michel Chonchol, Kristen L. Nowak

**Affiliations:** Division of Renal Diseases and Hypertension, University of Colorado Anschutz Medical Campus, Aurora, CO, United States

**Keywords:** chronic kidney disease, vascular health, lifestyle interventions, exercise training, inspiratory

## Abstract

Patients with chronic kidney disease (CKD) demonstrate accelerated vascular aging which contributes to an increased risk of cardiovascular disease (CVD). Impaired vascular health in CKD is characterized by both functional and structural alterations to the vasculature including hypertension, arterial stiffness, vascular endothelial dysfunction, and autonomic dysfunction. These detriments persist despite pharmacological intervention. Habitual aerobic exercise can be protective of vascular health; however, the feasibility in patients with CKD is low due to numerous barriers to exercise. In this perspective we emphasize the need for novel and non-pharmacological strategies that can rescue vascular health and reduce the development of CVD in patients with CKD, explain the unique barriers to aerobic exercise in CKD, present a novel physical training intervention—high-resistance inspiratory muscle strength training (IMST) that addresses the barriers to exercise, and provide our opinion on why this lifestyle intervention may be particularly efficacious for patients with CKD.

## 1 Introduction

Chronic kidney disease (CKD) is a multifaceted condition involving cardiovascular complications and numerous cardiovascular disease (CVD) risk factors (Zoccali et al., 2023). Moreover, individuals with CKD are more likely to die of CVD, than to progress to end-stage kidney disease (Sarnak et al., 2003). Patients with CKD present with an accelerated vascular aging phenotype characterized by functional and structural alterations that have been implicated in CVD development (Hobson et al., 2023). Healthy lifestyle practices such as habitual exercise can improve vascular health. However, patients with CKD have numerous barriers to implementing exercise. A novel physical training intervention, high-resistance inspiratory muscle strength training (IMST) can reduce barriers to exercise for patients with CKD and has demonstrated health benefits for older adults and patients with chronic disease. This perspective will review the impairments in vascular health in patients with CKD and propose why this novel mode of physical training, high-resistance IMST, is a viable strategy to improve vascular health, and thus, reduce the risk of CVD in patients with CKD.

### 1.1 Chronic kidney disease

CKD is defined as kidney damage and/or reduced kidney function present for at least 3 months (Levey et al., 2005). Kidney damage and impaired function can be confirmed by presence of albumin in the urine (albumin to creatinine ratio above 30 mg/g) and/or an estimated glomerular filtration rate (GFR) of less than 60 mL/min/1.73 m^2^, respectively (Levey et al., 2005). The lifetime risk of developing a GFR of less than 60 mL/min/1.73 m^2^ is above 50% (Grams et al., 2013), and CKD affects over 275 million individuals worldwide (Xie et al., 2018). CKD is typically not a single disease but a manifestation of multiple diseases that induce kidney damage acquired due to genetic and environmental or lifestyle factors.

**FIGURE 1:**
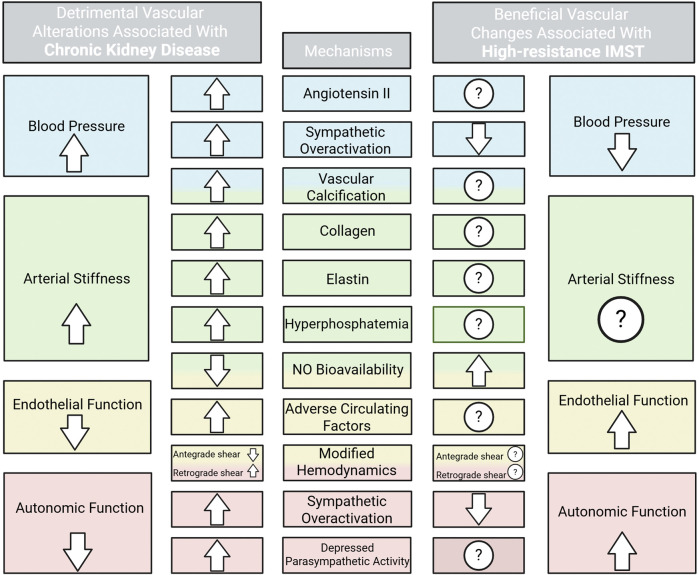
High blood pressure, arterial stiffness, and impaired endothelial and autonomic function are contributors to cardiovascular disease development in chronic kidney disease. The physiological stressors contributing to each vascular detriment and the current evidence and potential for IMST to improve these stressors are shown. ROS, reactive oxygen species. Created in BioRender. Lapierre-Nguyen, S. (2025) https://BioRender.com/w7vy2r8.

The incidence and prevalence of CVD remains high in patients with CKD despite the use of pharmacological therapies. Additionally, given the high pill burden in these patients (Oosting et al., 2024), novel, non-drug therapeutic approaches are warranted to ameliorate vascular health. Both functional and structural abnormalities of vascular function are contributors to CVD development in CKD (Figure 1). Impaired renal function influences vascular dysfunction by promoting a uremic circulating milieu and amplifying physiological stressors such as oxidative stress, inflammation, hyperphosphatemia, vascular calcification, and activation of angiotensin II (Kooman et al., 2014). Altered vascular health in patients with CKD present as hypertension (Ku et al., 2019), arterial stiffness (Briet et al., 2006), endothelial dysfunction (Ghiadoni et al., 2004), and autonomic dysfunction (Grassi and Drager, 2024) which collectively remodel the vasculature and exacerbate dysfunction of the cardiovascular system.

## 2 Diminished vascular health in CKD

### 2.1 Hypertension

The Systolic Blood Pressure Intervention Trial (SPRINT) was a landmark, multi-center randomized controlled trial comparing intensive versus standard blood-pressure control in adults with systolic blood pressure of 130 mmHg or higher. The trial found overwhelming evidence that intensive systolic blood pressure control significantly reduces the risk of cardiovascular and all-cause mortality in both the overall cohort and a CKD subgroup, without harmful effects on kidney function (Cheung et al., 2017; Wright et al., 2015). The SPRINT trial was the main factor leading to reclassification of hypertension guidelines by the American College of Cardiology (ACC) and American Heart Association (AHA) in 2017. In addition to the results from SPRINT, two large meta-analyses of patients with CKD reported that more intensive blood pressure control resulted in a 14% lower risk of all-cause mortality (Malhotra et al., 2017) and delayed the need for kidney replacement therapy (Ku et al., 2023). These studies prompted the Kidney Disease: Improving Global Outcomes (KDIGO) Blood Pressure Work Group to also adjust their hypertension treatment goals for CKD to a target systolic blood pressure of less than 120 mmHg (Cheung et al., 2021). Currently ACC/AHA define hypertension as stage 1, representing systolic blood pressure between 130 and 139 mmHg or diastolic blood pressure between 80 and 90 mmHg, and stage 2— at or above 140 mmHg systolic and 90 mmHg diastolic, with systolic blood pressure 120 mmHg and above considered as elevated (Whelton et al., 2017a).

Hypertension is highly prevalent (exceeding 75%) in patients with CKD, which is 30% greater than individuals without CKD (United States Renal Data System, 2023). Hypertension in patients with CKD is both a consequence of CKD and a driver of renal dysfunction and damage (Bidani and Griffin, 2002; Neuringer and Brenner, 1993). Systolic blood pressure predicts end-stage renal disease, coronary heart disease, and stroke, while diastolic blood pressure is not associated with vascular outcomes in patients with CKD (Kovesdy et al., 2016). Thus, targeting elevated systolic blood pressure is a focus of treatment. Despite the established clinical goal to lower systolic blood pressure in CKD, few patients attain controlled blood pressure. Approximately 50%–70% of adults with CKD have uncontrolled blood pressure despite treatment with multiple antihypertensive medications (Horowitz et al., 2015). Importantly, hypertension is influenced by numerous systemic and vascular components (i.e., arterial stiffness, decreased nitric oxide [NO] bioavailability, autonomic dysregulation). Thus, the redundancy of factors contributing to hypertension in CKD can be overwhelming and difficult to treat using solely pharmacological approaches.

### 2.2 Arterial stiffness

Large elastic arteries serve as conduits for the cardiovascular system, delivering blood and thus nutrients to tissues and organs. However, arteries have an additional fundamental function—a dampening effect which enables continuous blood flow into capillaries and reduces pressure in the microcirculation (O'Rourke and Franklin, 2006). Healthy elastic arteries must be distensible to expand in systole and recoil in diastole, which maintains blood flow during the cardiac cycle and functions to buffer the large pressure wave produced by left ventricular contraction (O'Rourke and Franklin, 2006). Epidemiological data indicate arterial stiffness is an independent risk factor for CVD (Niiranen et al., 2016), is associated with decreased GFR (Briet et al., 2006; Wang et al., 2005), and predicts kidney disease progression (Wang et al., 2005).

Large elastic artery stiffening occurs with advancing age (Vaitkevicius et al., 1993), but to a greater magnitude in patients with CKD (Nowak et al., 2020). Large artery stiffening is in part a consequence of structural changes in the vascular wall such as increased media thickness, increased collagen, reduced elastin, and calcification (Lakatta, 2003). Arterial stiffness is also determined by functional components, such as endothelial regulation of vascular smooth muscle tone (Lakatta, 2003). Impaired endothelial function is commonly a precursor of numerous chronic diseases known to also have increased arterial stiffness (Gimbrone, 1999). Thus, an interaction likely occurs between the development of endothelial dysfunction and altered structural components of the vascular wall, which in turn contributes to arterial stiffness. This relationship is demonstrated by transplantation studies, arterial stiffness is improved following kidney transplant as soon as 1 day after surgery, indicating a functional (non-structural) improvement (Kovács et al., 2013). Progressive improvements in arterial stiffness occur up to 1-year post-transplant (Keven et al., 2008; Ignace et al., 2011; Hotta et al., 2012) and likely results from both functional and structural changes. Additionally, there is a close relationship between arterial stiffness and systolic blood pressure (Mitchell, 2014). Elevated blood pressure may accelerate structural changes in the vascular wall, and greater arterial stiffness in normotensive individuals is associated with both renal dysfunction and future development of hypertension; thus, elastic artery stiffening may precede hypertension (Dernellis and Panaretou, 2005).

### 2.3 Vascular endothelial dysfunction

Endothelial dysfunction is a term representing impaired processes of the vascular endothelium that are involved in atherogenesis. The vascular endothelium consists of a single layer of cells forming the interface between the blood vessel wall and blood flow in the lumen. A healthy endothelium mediates endothelium-dependent vasodilation (largely determined by NO bioavailability), and inhibits thrombosis and coagulation, vascular inflammation, and vascular hypertrophy (Landmesser et al., 2004). Endothelial dysfunction is present in coronary arteries of individuals with advanced atherosclerosis (Ludmer et al., 1986) and independently predicts cardiovascular events (Halcox et al., 2002). Additionally, peripheral endothelium-dependent vasodilation assessed using flow-mediated dilation (FMD) is prognostic of cardiovascular events (Inaba et al., 2010). Patients with CKD exhibit impaired endothelium-dependent dilation assessed as brachial artery FMD (Ghiadoni et al., 2004; Thambyrajah et al., 2000). Impaired endothelial function can also occur through actions of endothelin, a powerful vasoconstrictor released from endothelial cells (Maguire and Davenport, 1995). Endothelin attenuates NO-mediated dilation through production of reactive oxygen species and levels of endothelin are inversely correlated to eGFR (Dhaun et al., 2015; Thengchaisri et al., 2015). The presence of endothelial dysfunction contributes to the functional component of arterial stiffness and hypertension which further exacerbates vascular dysfunction in CKD.

### 2.4 Autonomic dysfunction

The autonomic nervous system involves balance between sympathetic and parasympathetic outflow. Dysfunction is characterized by an increase in sympathetic activity and depressed parasympathetic activity (Pal et al., 2013). Overactivation of the sympathetic nervous system increases cardiovascular risk and influences hypertension, arterial stiffness, and endothelial dysfunction (Grassi and Drager, 2024). Autonomic dysfunction has been implicated in the increased cardiovascular disease risk in patients with CKD. Patients with CKD have a chronic overactivation of the sympathetic nervous system demonstrated by greater resting muscle sympathetic nerve activity and plasma catecholamines compared to controls (Dell'Oro et al., 2021; Grassi et al., 2021). The magnitude of sympathetic overactivation worsens as kidney disease progresses (Grassi et al., 2011). Additional evidence indicates patients with CKD have reduced baroreflex sensitivity, which was associated with increased levels of calcium-phosphate product, increased blood pressure variability, and decreased heart rate variability (Lal et al., 2017). This demonstrates complex interactions of several vascular components and the autonomic nervous system in patients with CKD thus, interventions must robustly target systemic vascular dysfunction.

## 3 Exercise strategies to restore vascular health in CKD

The use of pharmacological therapy is intended to manage the complex and integrated nature of CKD and its complications. The prevalence of polypharmacy, defined as concomitant use of ≥5 medications, in patients with CKD is estimated to be above 80% (Oosting et al., 2024). Additionally, participants in the SPRINT trial needed an average of three blood pressure medications to achieve the blood pressure goal of less than 120 mmHg for systolic blood pressure (Cushman et al., 2022). Importantly, polypharmacy is associated with increased risk for all-cause mortality and CVD, decline in eGFR, and lower quality of life in patients with CKD, after adjusting for other risk factors (Oosting et al., 2024). Moreover, the high prevalence of resistant hypertension in CKD (Horowitz et al., 2015) places further importance on more novel and non-pharmacological strategies for reducing blood pressure and improving vascular function in CKD.

The updated guidelines for blood pressure management from the collective effort of the ACC/AHA emphasize lifestyle strategies, specifically aerobic exercise, as first-line, standard-of-care interventions for adults with all stages of hypertension (Whelton et al., 2017b). Meta-analyses support strong evidence for the efficacy of aerobic exercise training to lower blood pressure, with the largest effects observed in adults with higher baseline systolic blood pressure (Whelton et al., 2002). Aerobic exercise training has beneficial effects on vascular function in middle-aged and older adults and individuals with chronic disease (Santos-Parker et al., 2014; Miele and Headley, 2017; Wang et al., 2022). KDIGO recommends a combination of lifestyle modifications and drug therapy to lower blood pressure, and specifically suggests 150 min per week of moderate intensity physical activity for individuals with CKD (Becker et al., 2012). A recent review summarized the effects of aerobic exercise interventions on vascular function in patients with CKD and concluded that higher-intensity aerobic exercise may be necessary to improve endothelial function, while the evidence is inconclusive for the effect on arterial stiffness (Kirkman and Chavez, 2024). The review emphasized the need to develop more practical exercise interventions for patients with CKD (Kirkman and Chavez, 2024). Despite the strong potential for improving vascular health, adherence to regular physical activity declines progressively from 34% in patients with stages 1–2 CKD to less than 6% of patients with more advanced CKD (Wilkinson et al., 2019; Robinson-Cohen et al., 2013).

### 3.1 Barriers to regular exercise in CKD

Chronic kidney disease presents unique challenges to lifestyle interventions and regular exercise. Physical barriers that limit the adoption of regular exercise include CKD-related symptoms such as mobility issues and frailty (Walker et al., 2013), fatigue and shortness of breath (Delgado and Johansen, 2012), and joint pain (Abdel-Kader et al., 2009), which collectively lead to poor physical function that is exacerbated by disease progression and comorbidities (Heiwe and Jacobson, 2011; Roshanravan et al., 2012). Mechanisms that have been implicated in the reduced physical condition of patients with CKD include impaired skeletal muscle mitochondrial metabolism (Conjard et al., 1995), reduced muscle protein synthesis (Adey et al., 2000), protein wasting (Wang and Mitch, 2014), and chronic inflammation (Wang et al., 2023).

Logistical barriers also play a major role in the lack of regular exercise in patients with CKD. These include lack of time and inaccessibility to facilities due to financial costs and mobility or transportation issues (Roshanravan et al., 2017). A recent study observed that 73% of patients with CKD indicated their preferred exercise location was at home (Moorman et al., 2019). Moreover, being able to exercise close to home is a key facilitator to implementing regular exercise in patients with CKD (Parsons et al., 2018). Thus, the utility of home-based exercise interventions, particularly in CKD should not be undervalued. Bringing aerobic exercise into the home can address one major barrier as far as accessibility; however, other psychological barriers may remain, such as fear of injury and CKD aggravation, low motivation, depression, and poor exercise self-efficacy (Clarke et al., 2015). Aerobic exercise is not a viable non-pharmacological intervention for the majority of patients with CKD due to its inability to reduce psychological barriers and by adding further burden of logistical and physical obstacles to patients. Thus, novel non-pharmacological interventions that overcome CKD-specific barriers are a critical need for patients with CKD.

### 3.2 High-resistance inspiratory muscle strength training

**FIGURE 2:**
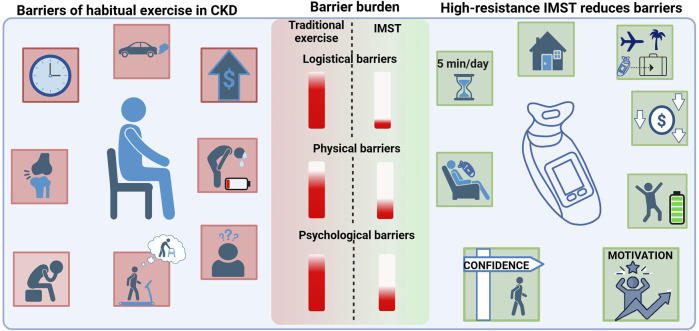
The unique barriers to regular exercise in patients with CKD (left) include lack of time and accessibility to exercise (logistical barriers), poor physical function including joint pain, fatigue and shortness of breath (physical barriers), and low motivation, depression, fear of injury, and poor exercise self-efficacy (psychological barriers). High-resistance IMST reduces numerous barriers to exercise in patients with CKD (right). High-resistance IMST can be conducted at home or while traveling due to the device being portable. Additionally, it is low-cost and can be completed safely in only 5 min per day. High-resistance IMST may improve fatigue and enhance patient self-efficacy and motivation to complete exercise. The burden of each type of barrier is indicated by the red bar. Logistical and psychological barriers present the highest burden, and IMST has potential to greatly reduce those barriers. Created in BioRender. Lapierre-Nguyen, S. (2025) https://BioRender.com/15q46cp.

High-resistance inspiratory muscle strength training (IMST) is a time-efficient and novel mode of physical training. High-resistance IMST is a form of resistance and muscular strength training that involves using the diaphragm and accessory respiratory muscles to inhale against resistance set by the handheld device. To inhale, an individual must generate large negative intrathoracic pressures, and exhaling is unimpeded. High-resistance IMST is a promising intervention, particularly for patients with CKD, as it addresses numerous barriers to exercise interventions (Figure 2). The IMST device is affordable, small, and portable; thus, the training can be performed at home or during travel. This form of physical training also overcomes barriers related to frailty, mobility disability, exercise intolerance, cost, and psychological barriers such as fear of falling or injury. High resistance-IMST has been successfully implemented in patient groups with common comorbidities to CKD these include generally healthy midlife and older adults with elevated blood pressure (Craighead et al., 2021a), individuals with obstructive sleep apnea (Vranish and Bailey, 2016; Ramos-Barrera et al., 1985), patients with chronic obstructive pulmonary disorder (Sanchez Riera et al., 2001; Preusser et al., 1994), and patients with type 2 diabetes (Reed et al., 2024). A commonly used protocol for high-resistance IMST includes subjects performing 30 breaths per session against an inspiratory resistance of 75% of maximal inspiratory pressure (PI_max_) (Craighead et al., 2021a; Vranish and Bailey, 2016; Ramos-Barrera et al., 1985; DeLucia et al., 2018; Vranish and Bailey, 2015). This protocol can be completed in approximately 5 minutes per day, and when performed 5–7 times per week, it amounts to a total training time of 30 min per week. Notably, adherence to this specific protocol has been remarkable (greater than 90% of prescribed training sessions were performed) (Vranish and Bailey, 2016; DeLucia et al., 2018; Vranish and Bailey, 2015; Craighead et al., 2021b).

High-resistance IMST confers similar benefits to aerobic exercise, while accomplishing these benefits in significantly less time (Craighead et al., 2024). In generally healthy midlife and older adults with above-normal systolic blood pressure, 6 weeks of high-resistance IMST increases exercise tolerance and favorably influences regional lean (increased thorax lean mass) and fat (reduced trunk fat mass) (Craighead et al., 2024). The increased exercise tolerance and thorax lean mass may be induced by thickening of the diaphragm as demonstrated in a healthy adults following 8 weeks of high-resistance IMST (Enright et al., 2006). High-resistance IMST reduces both casual and ambulatory systolic blood pressure in young, healthy adults, adults with obstructive sleep apnea, and midlife and older adults with above-normal systolic blood pressure (Vranish and Bailey, 2016; Ramos-Barrera et al., 1985; DeLucia et al., 2018; Craighead et al., 2021b). Although, guidelines for management of hypertension focus on systolic blood pressure to inform the initiation of antihypertensives and for calculating risk of adverse cardiovascular events, high-resistance IMST is also efficacious at modestly reducing casual diastolic blood pressure as demonstrated by a retrospective analysis of five studies utilizing high-resistance IMST (Craighead et al., 2022). The efficacy of high-resistance IMST for improving vascular function has been equivocal, but this is likely due to differences in populations being studied and the degree of vascular dysfunction at baseline (Craighead et al., 2021a). One promising investigation in midlife and older adults with above-normal blood pressure found significant improvements in brachial artery endothelial function after 6 weeks of high-resistance IMST, but not after Sham training (15% PI_max_) (Craighead et al., 2021b). There was not a concurrent reduction in arterial stiffness, but structural changes that influence large elastic artery stiffening can require interventions of longer duration (Pierce, 2017). These improvements in blood pressure and endothelial function following high-resistance IMST in midlife and older adults with elevated blood pressure suggest this behavior may also be effective for patients with CKD, a patient group with similar age and likely worse blood pressure profiles.

## 4 The potential of IMST for improving vascular health in CKD

One particularly exciting aspect of the clinical trial conducted by Craighead and colleagues is that the systolic blood pressure lowering effect of only 6 weeks of high-resistance IMST persisted for 6 weeks after cessation of the intervention (Craighead et al., 2021b). This lasting effect contrasts with aerobic exercise, in which the effects are typically short-term and require continual effort (Mora-Rodriguez et al., 2014; Nolan et al., 2018). This factor alone is potentially very valuable for an effective lifestyle intervention for patients with CKD, as daily and weekly symptoms and motivation can vary.

We are currently conducting a randomized controlled trial (NCT04911491) investigating the efficacy of high-resistance IMST (75% PI_max_) versus Sham training (15% PI_max_) to reduce blood pressure and arterial stiffness and improve endothelial function, in midlife and older adults with stage 3–4 CKD. The trial is 12 weeks in length (30 breaths/session, 6x/week), which may induce greater benefits relative to previously completed 6-week interventions of high-resistance IMST. Several aspects of our trial that are particularly important for patients with CKD include: 1) the intervention is completed remotely allowing participants to complete the training when their schedule allows at home or while traveling, 2) a progressive ramping up to the training load during the first 2–4 weeks of training may increase patient self-efficacy and tolerance, 3) a combination of in-person and remote check-ins with experienced IMST exercise coordinators to provide real-time feedback and coaching, promoting adherence and providing technique adjustments to increase the percentage of successful breaths, 4) assessing PI_max_ every 2–3 weeks allows continuous load adjustment to maintain an appropriate training stimulus as participants adapt, and 5) the required time commitment is ∼5 min per day with a total training time of ∼30 min per week. We are still actively enrolling; however, completed participants have expressed they feel less fatigued, have improved breathing, and motivation to continue training following their study participation. These remarks are meaningful, as they indicate high-resistance IMST is a lifestyle behavior that patients with CKD could potentially adhere to long-term. Below we will briefly discuss evidence of why high-resistance IMST may be specifically effective in improving vascular health in patients with CKD.

### 4.1 Changes in the circulating milieu

The CKD circulating milieu consists of circulating factors that interact closely with the vascular wall and can induce maladaptive changes. Circulating factors that are typically altered in CKD and known to have detrimental vascular effects include reactive oxygen (ROS) species, proinflammatory molecules, and uremic toxins (Meyer and Hostetter, 2007). Markers of oxidative stress and proinflammatory molecules are increased in the circulation of patients with CKD (Yilmaz et al., 2006; Yilmaz et al., 2011). Uremic toxins are positively correlated with markers of oxidative stress in patients with CKD (Dou et al., 2015), and incubation of cultured human endothelial cells with a uremic toxin, indoxyl sulfate, influences ROS, NO production, and inflammation (Tumur and Niwa, 2009; Tumur et al., 2010). Moreover, previous work from our group has shown acute intravenous infusion of vitamin C does not rescue endothelium-dependent dilation in patients with CKD, indicating the high degree of vascular oxidative stress present in these patients cannot be overcome with the addition of a circulating antioxidant (Nowak et al., 2020). Thus, vascular oxidative stress is a main determinant of endothelial dysfunction in patients with CKD.

High-resistance IMST may oppose adverse factors in the CKD circulating milieu. A new and novel method has been employed to investigate the role of individuals’ circulating milieu on vascular function by using an *ex vivo* approach. This method entails incubating cultured human endothelial cells with serum from participants and subsequently assessing markers of endothelial cell function (Mahoney et al., 2024). The aforementioned trial conducted in midlife and older adults with above-normal systolic blood pressure found that incubation of human umbilical vein endothelial cells with participant serum following high-resistance IMST had significantly greater endothelial cell NO production and substantially less ROS activity compared to baseline and relative to the Sham training group (Craighead et al., 2021b). The finding of increased NO production persisted when human brain endothelial cells were used, indicating the large impact that the circulating milieu can have on various endothelial cell types (Freeberg et al., 2023). Additionally, systemic inflammation was reduced, reflected by reduced plasma CRP and increased hexanoic acid, a short-chain fatty acid that is involved in anti-inflammatory signaling (Gawron-Skarbek et al., 2023). High-resistance IMST also modified two other plasma metabolites that are involved in substrate availability for NO and blood pressure control, key modulators of vascular function. If similar changes in circulating factors with high-resistance IMST are observed in patients with CKD, this would be particularly impactful, as inflammation and oxidative stress are hallmarks of both accelerated vascular aging and CKD. The potential for changes in circulating uremic toxins with high-resistance IMST is currently unknown; however, in a meta-analysis, traditional, whole body resistance exercise, but not aerobic exercise training reduces plasma homocysteine (Deminice et al., 2016), a known uremic toxin (Perna et al., 1999). Thus, the efficacy for high-resistance IMST in improving circulating factors involved in oxidative stress and chronic systemic inflammation in patients with CKD is promising.

### 4.2 Modified hemodynamics

Exercise-induced changes (acute and long-term) in shear patterns are a primary mechanism contributing to chronic improvements in endothelial function, assessed as FMD (Tinken et al., 2010). Acute changes in shear stress profiles during and immediately after a session of IMST have been observed in young healthy adults. During both low- and high-resistance IMST, blood flow and shear rate in the upper and lower limbs are reduced (Plouffe et al., 2023). This finding was confirmed during a moderate resistance (50% of PI_max_) IMST session; antegrade blood flow and shear rate are reduced, while retrograde blood flow and shear rate are substantially increased during the inhalation phase compared to baseline (Tavoian et al., 2024). Directly following the session of IMST, FMD is improved and returns to baseline within 40-min of recovery. Interestingly, the magnitude of improvement in FMD is moderately correlated with the degree of increased retrograde shear rate during the session (Tavoian et al., 2024). Oscillatory shear patterns during high-resistance IMST may act as a stimulus for vascular remodeling (Tavoian et al., 2024). Moreover, even greater oscillatory shear patterns may occur with high-resistance IMST in populations known to have greater resting oscillatory blood flow. Importantly, these acute changes are a beneficial stimulus that likely induce adaptations for improved vascular function, while sustained maladaptive changes in shear (i.e., reduced antegrade shear rate and increased retrograde shear rate) are known to promote vascular pathologies. We recently have shown that patients with CKD have altered resting brachial artery shear patterns compared to age-matched controls, which likely plays a role in CKD-related endothelial dysfunction assessed as FMD (Lapierre-Nguyen et al., 2025). Thus, exploring changes in shear patterns during and directly following acute IMST, and after a sustained intervention of IMST in patients with CKD may provide information on mechanisms and potential targets for interventions to improve vascular function.

### 4.3 Decreased sympathetic nervous system activity

Sympathetic nervous system overactivity can increase blood pressure and promote vascular structural and functional changes that influence arterial stiffness (Nardone et al., 2020; Bruno et al., 2012). Reducing resting sympathetic nervous system activity is another potential mechanism by which high-resistance IMST may improve vascular function in patients with CKD. Muscle sympathetic nerve activity decreases in healthy young adults during and directly following an acute session of high-resistance IMST, which persists for 5 min into recovery (DeLucia et al., 1985). This study complements previous evidence of a similar acute reduction in sympathetic activity using lower inspiratory pressures in generally healthy adults (St Croix et al., 2000; St Croix et al., 1999). High-resistance IMST reduces muscle sympathetic nerve activity, circulating catecholamines, and blood pressure in older adults with obstructive sleep apnea (Vranish and Bailey, 2016; Ramos-Barrera et al., 1985) and lowers systemic vascular resistance and blood pressure in young healthy adults (DeLucia et al., 2018). In contrast, plasma catecholamines are unchanged in a similar trial in healthy midlife and older adults with above-normal blood pressure (Craighead et al., 2021b). These differences may be due to plasma catecholamines being an indirect measure of sympathetic nerve activity relative to using microneurography to assess muscle sympathetic nerve activity. Effects of high-resistance IMST on reducing sympathetic activity may be more evident in patients with CKD, as demonstrated by an 8-week moderate intensity IMST intervention that lowered cardiac sympathetic modulation in patients with type 2 diabetes (Kaminski et al., 2015), one of the leading causes of CKD.

## 5 Conclusion

A common theme in the field of exercise physiology is the age-old question of the best physical activity mode, duration, and intensity for health benefits. In closing, we’d like to emphasize the most beneficial lifestyle intervention in patients with CKD is the one that patients can and will adhere to. Thus, we believe high-resistance IMST is a promising strategy for patients with CKD as it reduces many of the barriers to exercise with the potential of inducing lasting effects on vascular health. An accessible and effective method for improving vascular health would be highly impactful for reducing the elevated risk of CVD in this rapidly growing patient population.

## Data Availability

The original contributions presented in the study are included in the article/supplementary material, further inquiries can be directed to the corresponding author.
